# Comparison of Methicillin Resistant *Staphylococcus Aureus* in Healthy Community Hospital Visitors [CA-MRSA] and Hospital Staff [HA-MRSA]

**DOI:** 10.4084/MJHID.2015.053

**Published:** 2015-10-07

**Authors:** Nirmal A Pathare, Sara Tejani, Harshini Asogan, Gaitha Al Mahruqi, Salma Al Fakhri, Roshna Zafarulla, Anil V. Pathare

**Affiliations:** 1 Oman Medical College, Muscat, Oman; 2 Sultan Qaboos University Hospital, Muscat, Oman

## Abstract

**Background:**

The prevalence of community-associated methicillin-resistant *Staphylococcus aureus* [CA-MRSA] is unknown in Oman.

**Methods:**

Nasal and cell phones swabs were collected from hospital visitors and health-care workers on sterile polyester swabs and directly inoculated onto a mannitol salt agar containing oxacillin, allowing growth of methicillin-resistant microorganisms. Antibiotic susceptibility tests were performed using Kirby Bauer’s disc diffusion method on the isolates. Minimum inhibitory concentration (MIC) was determined for vancomycin and teicoplanin against the resistant isolates of MRSA by the Epsilometer [E] test. A brief survey questionnaire was requested be filled to ascertain the exposure to known risk factors for CA-MRSA carriage.

**Results:**

Overall, nasal colonization with CA-MRSA was seen in 34 individuals (18%, 95% confidence interval [CI] =12.5%–23.5%), whereas, CA-MRSA was additionally isolated from the cell phone surface in 12 participants (6.3%, 95% CI =5.6%–6.98%). Nasal colonization prevalence with hospital-acquired [HA] MRSA was seen in 16 individuals (13.8%, 95% confidence interval [CI] =7.5%–20.06%), whereas, HA-MRSA was additionally isolated from the cell phone surface in 3 participants (2.6%, 95% CI =1.7–4.54). Antibiotic sensitivity was 100% to linezolid and rifampicin in the CA-MRSA isolates. Antibiotic resistance to vancomycin and clindamycin varied between 9–11 % in the CA-MRSA isolates. Mean MIC for vancomycin amongst CA- and HA-MRSA were 6.3 and 9.3 μg/ml, whereas for teicoplanin they were 13 and 14 μg/ml respectively by the E-test. There was no statistically significant correlation between CA-MRSA nasal carriage and the risk factors (P>0.05, Chi-square test).

**Conclusions:**

The prevalence of CA-MRSA in the healthy community hospital visitors was 18 % (95% CI, 12.5% to 23.5%) as compared to 13.8% HA-MRSA in the hospital health-care staff. Despite a significant prevalence of CA-MRSA, these strains were mostly sensitive. Recommendation: The universal techniques of hand washing, personal hygiene and sanitation are thus warranted.

## Introduction

Methicillin resistant *Staphylococcus aureus* [MRSA] has emerged as a virulent pathogen and is a leading cause of nosocomial infections.^1^–^3^ Although first reported in 1972, there are increasing number of hospital outbreaks with increased mortality, morbidity and health care costs.^4^ Furthermore, since 1990s, MRSA has also emerged as cause of infection in the community.^5^,^6^ Community-acquired MRSA [CA-MRSA] is usually seen in subjects with well recognized risk factors such as intravenous drug usage, debilitating co-morbid conditions like diabetes mellitus, malignancies, cardiovascular or renal failure, etc.^7^–^11^ However, the first reported CA-MRSA was in Australian aboriginals and native Canadians in the 1990s^12^. The first reported cases of CA-MRSA in the USA were seen with no contact to health care system in native Americans from Minnesota, North Dakota and Nebraska as well as in Los Angeles and San Francisco.^13^,^14^

The emergence of CA-MRSA in the community is a significant public health concern as transmission from individual to individual is a primary health care concern leading to the spread of microorganisms of significant potential for morbidity and mortality. However, not the mere presence of CA-MRSA but its antibiotic sensitivity-resistance pattern plays a significant role in the risk assessment. Methicillin-resistant strains became more common than methicillin-susceptible strains, first in hospitals [HA-MRSA] and later in the community.^3^,^15^ Prevalence of CA-MRSA is variable, being low in some European countries, whereas, there is increasing evidence that it is significantly higher in many other parts of the world.^16^,^17^ Some studies from India have reported a prevalence of CA-MRSA in the range of 4.6–10.6% from a rural setting,^18^,^19^ whereas others like Gaud et al reported CA-MRSA prevalence of 16.4% in an urban setting from Bangalore, in India.^20^

Thus, in view of the rising trend of the increasing prevalence of CA-MRSA, and its propensity to develop resistance, it is imperative not only to study the prevalence of CA-MRSA in Oman, but also its antibiotic susceptibility and resistance pattern. Unfortunately, the prevalence of CA-MRSA in Oman is unknown.

## Aim of this Study

We initiated this study to screen for CA-MRSA by per nasal and cell phone swabs as it will give an additional perspective to the Omani health initiative. Participants were also asked to fill a brief survey questionnaire to record their age, gender, history of infections, if any, frequency of hospital visits, as well as the associated co-morbid conditions like diabetes, hypertension or recent skin/wound infections and antibiotic exposure.

## Patients and Methods

Study design and subjects: The study design was a prospective cross-sectional cohort study and was approved by the institutional research and ethics committee. Participants were enrolled after a written informed consent. The hospital visitor community and health-care workers from the hospital, as well as private clinics, were enrolled in the study. Nasal and cell phones swabs from all participants were inoculated onto a selective mannitol salt agar with oxacillin with minimum time lapse that would allow growth of only methicillin-resistant microorganisms. Demographic data collected included age, gender, and nationality. To assess any possible risk for MRSA carriage, further data including hospital exposure, exposure to antibiotics, co-morbidities like diabetes mellitus, hypertension and skin and soft tissue wounds, etc. was also collected and recorded.

Sample collection and transportation: Samples were collected from both anterior nares using sterile polyester swabs with a standard rotating technique. Similarly, the surface of participant’s personal cell phones were also swabbed using swabs pre-moistened with sterile saline.^21^ These swabs were used for inoculation of mannitol salt agar containing oxacillin with minimum time lapse. Colony characteristics on the culture plates and Gram-staining were used to confirm further the identity of *Staphylococcus aureus* that grew on this MRSA selective medium. Gram staining helped to ascertain that there were no other airborne contaminants by confirming the characteristic morphology of *Staphylococcus aureus*. Growth of any other microorganisms were noted but was not included in this analysis.

Antibiotic susceptibility tests were performed on Mueller-Hinton agar using Kirby Bauer’s disc diffusion method,^22^ according to the Clinical and Laboratory Standards Institute (CLSI) guidelines. *S. aureus* ATCC 25923 was used as a control strain. The following antibiotics were used: erythromycin (15 μg), clindamycin (2 μg), rifampicin (2 μg), doxycycline (30 μg), vancomycin (30 μg), linezolid (30 μg) and teicoplanin (30 μg). MIC for vancomycin and teicoplanin were further tested by the Epsilometer [E] test (Ezy MIC Strip, HIMEDIA India) against resistant MRSA strains. MIC values were read as per the manufacturers recommendation and interpretation made as per CLSI criteria.^23^

### Statistical Analysis

All the data was analyzed using IBM-SPSS ver 19.0. The prevalence of MRSA was estimated with 95% confidence intervals. Continuous variable are reported as mean ± SD with 95% Confidence Intervals. The correlation between categorical variables was determined by Chi-Square test for significance. Statistical significance was identified as p<0.05.

## Results

The hospital visitor community comprised of 189 subjects, with a mean age of 25.43±17.5 years old; most of the subjects were female (63.5%). The hospital health-care workers comprised of 116 subjects, with a mean age of 33.23±8.9 years old; the majority of the persons in this cohort were females (67.2%) (Table 1a). There was no statistically significant correlation between CA- and HA-MRSA isolates and the demographic characteristics or the risk factors namely gender, underlying co-morbidities like diabetes, hypertension, skin/soft tissue infections, skin ulcers/wounds, recent exposure to antibiotics, or hospital exposure (Table 1b;P>0.05, Chi-square test). Overall, in the hospital visitor community, CA-MRSA were isolated in 34 individuals from their nasal vestibules giving a carriage rate of 18.0% (95% CI =12.5% to 23.5%). (Table 2) CA-MRSA was also isolated from the cell phone surfaces in 12 individuals yielding a carriage rate of 6.3% (95% CI =5.6% to 6.98%). In 2 participants (1.06%), CA-MRSA was isolated both from nasal vestibules and from their cell phone swabs. In the hospital health workers, the nasal carriage [HA-MRSA] was observed in 16 individuals with a colonization rate of 13.8% (95% CI =7.5% to 20.06%) and in 3 individuals, cell phone swabs grew HA-MRSA giving a colonization rate of 2.6% (95% CI =1.7% to 4.54%). However, none of the hospital health workers showed positive MRSA isolation from nose and cell-phone from the same individual. A total of 46 and 19 isolates were respectively obtained and confirmed as CA-MRSA and HA-MRSA from the culture characteristics and Gram staining in the community hospital visitors and the hospital health-care workers. Amongst the CA-MRSA isolates antibiotic resistance with erythromycin and clindamycin varied between 11–35%, whereas, most isolates were sensitive to rifampicin, doxycycline, vancomycin, linezolid, and teicoplanin. (Figure 1a)

However, amongst the HA-MRSA isolates, a significantly higher antibiotic resistance was seen with both erythromycin and clindamycin, varying between 42–63%, whereas, the sensitivity of HA-MRSA isolates to rifampicin, doxycycline, vancomycin, and linezolid was 95%, 89%, 84%, and 100% respectively (Figure 1b). Overall, there was no significant differences in the resistance pattern between the nasal and cell phone CA-MRSA isolates [Table 3; p>0.05, Chi-square test] Overall, the vancomycin-resistant CA-MRSA were 2.1%. Mean MIC by the E test for vancomycin amongst CA-MRSA isolates [n=4] was 6.5 μg/ml with a range between 6 to 8, whereas, amongst the HA-MRSA isolates [n=3] it was 9.3 μg/ml with a range between 8 to 12. Mean MIC by the E test for teicoplanin amongst CA-MRSA isolates [n=4] was 13 μg/ml with a range between 12 to 16, whereas amongst the HA-MRSA isolates [n=4] it was 14 μg/ml with a range between 12 to 16.

## Discussion

The prevalence of MRSA in Oman is unknown. This study showed a relatively higher overall prevalence of CA-MRSA nasal carriage in an urban setting of 18% as compared to HA-MRSA nasal colonization of 13.8%. Thus, although the exposure of hospital environment should have led to an increased prevalence, yet the impact of ongoing hospital infection control policies and other related activities, like hand washing and personal hygiene, could have resulted in this relative lower prevalence over community CA-MRSA nasal carriage. Furthermore, the higher incidence of CA-MRSA amongst hospital visitors also makes it more likely to have been acquired in an out of hospital setting. This is in keeping with the fact that although CA-MRSA is known to be associated with co-morbidities such as diabetes mellitus, malignancies, cardiovascular or renal failure,^7^–^11^ is has been also reported in subjects with no contact to health care system.^13^,^14^ However, the more disconcerting fact is that the vancomycin MIC values for HA-MRSA by the E-test were much higher than those seen in the CA-MRSA resistant isolates. All this could have important implications in the setting of community-acquired nosocomial infections.

Literature review on the prevalence data of CA-MRSA shows an almost 2-fold increase in prevalence rates in our study in comparison to previous Indian studies reporting a prevalence of CA-MRSA to range between 4.6% to 10.6%^18^,^19^. However these reports were from a rural setting in the Indian state of Karnataka, whereas, Gaud et al^20^ reported a CA-MRSA prevalence of 16.4% from an urban setting in Bangalore, from India. Therefore, it seems that rural CA-MRSA prevalence is much lower than the urban setting as corroborated by the prevalence data from our study from an urban setting.

In contrast, the prevalence of cell phone carriage of HA-MRSA was almost half of the CA-MRSA at 2.3% v/s 6.3% in our study. Lower cell phones carriage of HA-MRSA was especially important as it indicates the need to reinforce an awareness campaign in the community to take adequate care and precautions regarding the universal techniques of hand washing, personal hygiene, and sanitation. Moreover, contaminations of inanimate objects like cell phones should also be reduced to minimize the risk of transmitting CA-MRSA as these organisms are generally spread by a person to person transmission. Furthermore, the incidence and nature of antibiotic resistance patterns of CA-MRSA will be a pivotal issue for further risk assessment and management of these isolates.

Although *Staphylococcus aureus* is a commensal organism, it is the most common cause of skin and soft tissue infections and nosocomial infections and antibiotic resistance is an ever increasing concern. Furthermore, an outcome of these events is often pneumonia, wound sepsis, arthritis, endocarditis, or osteomyelitis leading to an increasing morbidity and mortality.^11^,^12^ The antimicrobial susceptibility profile of *S. aureus* and MRSA usually differs depending on the local settings, as seen in several reports depending on the resistance profiles observed.^3^–^5^,^8^,^19^,^21^,^22^ In this study, antibiotic susceptibility tests revealed that a majority of the CA-MRSA isolates were sensitive to most of the commonly prescribed antibiotics (Figures 1a and 1b), and especially as compared to HA-MRSA isolates. However, there was a significantly high rate of resistance to erythromycin [63%], clindamycin [42%] and Teicoplanin [21%] amongst the HA-MRSA isolates as compared to CA-MRSA isolates of 35%, 11% and 9% respectively. Moreover, it was also noticed that most of this resistance was associated with the per nasal isolates amongst the HA-MRSA isolates (Table 3). Most of the HA-MRSA and CA-MRSA isolates were sensitive to rifampicin [95 v/s 100%], doxycycline [89 v/s 94%], vancomycin [84 v/s 91%] and linezolid [100% in both]. Only one strain of HA-MRSA showed resistance to rifampicin and was isolated from the nasal swab in a health care worker. The four strains of vancomycin resistance in the CA-MRSA isolates were equally distributed between the nose and cell phones. However, all the three strains of vancomycin resistance seen in the HA-MRSA isolates were obtained from a nasal swab of the hospital health-care staff, and this is a worrisome issue. Overall, the relatively higher prevalence of resistant HA-MRSA isolates amongst the hospital health-care staff is significant and needs to be addressed by the respective hospital infection control committees and protocols. It is prudent that immediate action is required to reduce the prevalence of these asymptomatic subjects with resistant HA-MRSA nasal colonization to protect unsuspecting and unfortunate hospital patients from being passively exposed and being at risk of possible transmission of nosocomial MRSA organisms. MRSA nosocomial infection outbreaks can also be thus prevented by appropriate immediate action and following the recommended infection control guidelines in the matter in these few identified cases with resistant MRSA nasal colonization. Fortunately, the study did not find any multi-drug resistant HA-MRSA isolates.

Although no risk factors were identified when CA-MRSA was initially reported,^13^,^14^ several risk factors are generally associated with HA-MRSA isolates^12^,^23^. In this study, we also explored several demographic as well as co-morbid risk factors, but there were no statistically significant correlations between HA-MRSA and CA-MRSA isolates and the risk factors. Specifically, we could not demonstrate any association between isolates of HA- and CA-MRSA and an underlying risk factor for diabetes, hypertension, skin/soft tissue infection, skin ulcer/wound, recent exposure to antibiotic, or hospital visit, etc.(P>0.05, Chi-square test).

In summary, the prevalence of asymptomatic nasal carriage of CA-MRSA was higher than noted in several previous reports involving community studies. No risk factors were significantly associated with this high prevalence. Although limited by the small sample size, a majority of the CA-MRSA isolates were relatively sensitive as compared to the HA-MRSA isolates. The very fact that prevalence of cell phone carriage in the community cohort is substantially higher compared to the health care worker cohort is a public health concern. It is therefore recommended that universal measures of hand washing, personal sanitation and hygiene need utmost attention, especially in the community and awareness campaign programs need to be implemented robustly.

## Figures and Tables

**Figure 1a:**
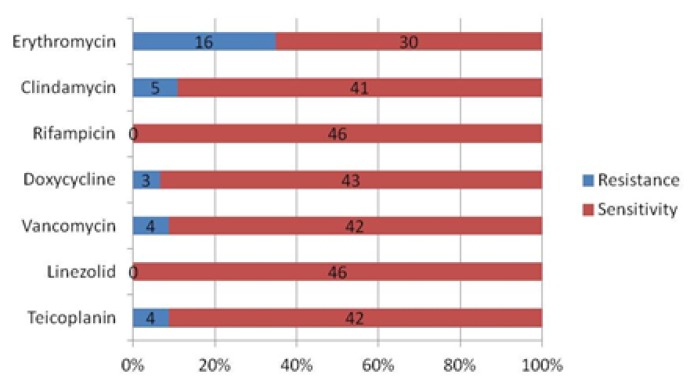
Antibiotic resistance and susceptibility pattern by the Kirby-Bauer Disc diffusion method in CA-MRSA isolated (n=46).

**Figure 1b:**
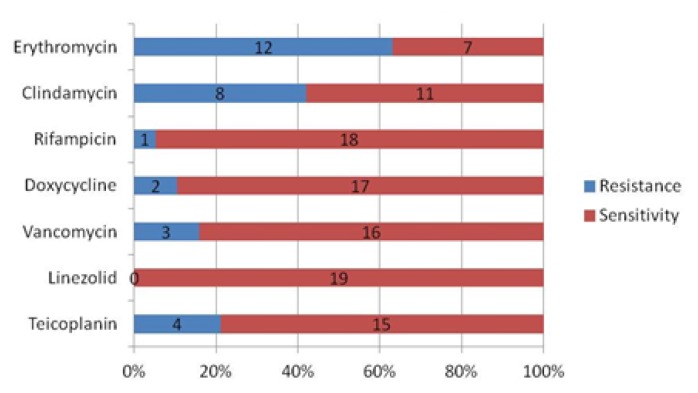
Antibiotic resistance and susceptibility pattern by the Kirby-Bauer Disc diffusion method in HA-MRSA isolated (n=19).

**Table 1a:** Demographic characteristics for the two study cohorts.

Characteristics	Number
**Community Cohort**, *n*	**189**
Male/Female	69/120
Mean age±SD, years	25.43 ± 17.5
Median Age, years	24
Range, years	1–85
**Hospital Health Care Cohort**, *n*	**116**
Male/Female	38/78
Mean age ± SD, years	33.23 ± 8.9
Median Age, years	30
Range, years	22–62

**Table 1b:** Underlying risk factors for the two cohorts of the study.

	Community Cohort [n=189]		Hospital Health Care Cohort[n=116]
Co-morbidities, %			
Diabetes Mellitus	5.0		6.5‡
Hypertension	4.4		6.5‡
Ulcers	0.6		0‡
Soft tissue infections	4.4		0.8‡
H/O Recent exposure to Antibiotics%	Yes	8.3	24.4‡
	No	71.7	75.6‡
H/o recent exposure/Hospitalization	Yes	1.1	3.3‡
	No	98.9	96.7‡
Presence of wounds/skin lesions	Yes	6.1	0.8‡
	No	93.9	99.2‡

‡ - NS, P>0.05, Chi Square test

**Table 2:** Prevalence of CA-MRSA and HA-MRSA isolates in the Community and Health Care staff.

Cohorts→		Community N=189	Hospital Health Care N=116	p value
Nasal	N, Prevalence [%]	34,[18]*	16,[13.8]	NS
	95% CI,	12.5–23.5	7.5–20.06	
Cell Phone	N, Prevalence [%]	12,[6.3]*	3,[2.6]	NS
	95% CI,	5.6–6.98	1.7–4.54	

CI- Confidence interval;

* NS, P>0.05, Chi Square test

**Table 3:** Antibiotic sensitivity and resistance pattern of the CA-MRSA [n=19] and HA-MRSA isolates [n=46].

Antibiotics	N^HA^#	CP^HA^#	R^HA^#	N^CA^#	CP^CA^#	R^CA^#	S^HA^†[%]	S^CA^†[%]
**Erythromycin[%]**	10[83]	2[17]†	12[100]	10[63]	6[37]*	16[100]	7[37]	30[65]*
**Clindamycin[%]**	7[88]	1[12]†	8[100]	3[60]	2[40]*	5[100]	11[58]	41[89]*
**Rifampicin[%]**	1[100]	0*	1[100]	0	0*	0	18[95]	46[100]*
**Doxycycline[%]**	2[100]	0*	2[100]	2[50]	1[50]*	3[100]	17[90]	43[94]*
**Vancomycin[%]**	3[100]	0*	3[100]	2[50]	2[50]*	4[100]	16[84]	42[91]*
**Linezolid[%]**	0	0*	0	0	0*	0	19[100]	46[100]*
**Teicoplanin[%]**	4[100]	0†	4[100]	3[75]	1[25]*	4[100]	15[79]	42[91]*

N- Nasal Swab isolates; CP- Cell phone Swab isolates;

# R-Number [%] of Resistant MRSA isolates;

-S^†^%-Number [%] of sensitive MRSA isolates, CA- Community hospital visitors; HA- Hospital Health-care workers;

† S - p<0.05, N v/s CP, Chi square test. ;

* NS - p<0.05, CA v/s HA, Chi square test.^†^
